# Targeting the IRE1α/XBP1 pathway in type 2 diabetes mellitus: from ER stress signaling to therapeutic opportunity

**DOI:** 10.1007/s11033-026-12876-7

**Published:** 2026-10-08

**Authors:** Olabisi Tajudeen Obafemi, Ademola Olabode Ayeleso, Bidemi Emmanuel Ekundayo, Blessing Ariyo Obafemi, Olusola Bolaji Adewale, Sogolo Lucky Lebelo, Monde Ntwasa

**Affiliations:** 1 Department of Life and Consumer Sciences, University of South Africa, Florida Campus, Johannesburg, 1709 South Africa; 2 Biochemistry Programme, College of Agriculture, Engineering and Sciences, Bowen University, Iwo, 232101 Nigeria; 3 Department of Biochemistry, Bamidele Olumilua University of Education Science and Technology, Ikere-Ekiti, Nigeria; 4 Department of Medical Biochemistry, Afe Babalola University, Km 8.5, Afe Babalola way, Ado-Ekiti 360001, Ekiti State, Nigeria; 5 Drosophila Research/Drug Metabolism and Toxicology Laboratories, Biochemistry Program, College of Sciences, Afe Babalola University, Km 8.5, Afe Babalola way, Ado-Ekiti 360001, Ekiti, Nigeria

**Keywords:** Drug target, X-box binding protein 1, Inositol-requiring enzyme 1-alpha, type 2 diabetes, conserved

## Abstract

To respond to protein misfolding in the endoplasmic reticulum (ER), cells trigger the unfolded protein response (UPR) to re-establish proteostasis. However, unresolved UPR results in chronic ER stress, which is critical to the development and advancement of type 2 diabetes mellitus (T2DM), a multifactorial disease whose prevalence has continued to rise. The UPR consists of three transmembrane signaling branches - protein kinase R-like ER kinase (PERK), activating transcription factor 6 (ATF6), and inositol-requiring enzyme 1 alpha (IRE1α), which collectively attempts to restore proteostasis, or trigger apoptosis if ER stress persists. Among the three, the IRE1α pathway is the most evolutionarily conserved and serves as a central regulator of UPR signaling through the splicing of X-box binding protein 1 mRNA (XBP1s). In the adaptive mode, XBP1s drives the expression of genes that expand ER capacity and alleviate cellular stress. Conversely, chronic activation of the IRE1α pathway activates the regulated IRE1-dependent decay (RIDD) process, recruits tumour necrosis factor receptor-associated factor 2/c-Jun N-terminal kinase (JNK), and NOD-, LRR-, and pyrin domain-containing protein 3 (NLRP3), with their attendant deleterious effects. Thus, the therapeutic utility of the IRE1α/XBP pathway depends on the duration and extent of UPR as well as tissue and cellular metabolic state. The extensive crosstalk of the pathway with metabolic and inflammatory pathways makes it an attractive therapeutic target. This review examines the role of the IRE1α/XBP1 pathway in T2DM and highlights the potential of modulating the pathway as a novel disease-modifying strategy in the context of the increasing burden of the disease.

## Introduction

Diabetes mellitus is a chronic metabolic disease whose major characteristic is persistent high blood glucose due to defective insulin secretion, defective insulin action, or both [1]. The number of diabetics reportedly increased from 200 million in 1990 to 830 million in 2022 [2]. The growing prevalence of the disease places massive burdens on healthcare systems, which highlights the pressing need to have better understanding of its pathogenic mechanisms [3]. Type 2 diabetes mellitus (T2DM), the class to which about 90–95% of diabetes cases belong, is characterized by insulin resistance accompanied by insufficient compensatory secretion of insulin [1]. The other types of diabetes are type 1 diabetes, which involves autoimmune-mediated destruction of pancreatic β-cells, leading to complete insulin deficiency, and gestational diabetes, whose main feature is chronic hyperglycemia that is initially diagnosed during the latter stages of pregnancy [4, 5]. Persistent hyperglycemia, which is the hallmark of untreated diabetes, leads to diabetic complications, as it progressively damages the vascular, renal, neurological, ocular, and cardiovascular systems, resulting in substantial morbidity, disability, premature mortality, and healthcare expenditure [2, 6].

Currently available antidiabetic drugs primarily target downstream metabolic processes, such as renal glucose reabsorption, insulin secretion, insulin sensitization, and direct insulin-release mechanisms, in order to achieve glycemic control, while limiting the physiological risks that may arise from modulating upstream pathways involved in broader cellular regulations [7–9]. Therapeutically targeting downstream signaling pathways provides relatively defined mechanisms of action and, in some cases, more glucose-centric regulation of blood sugar, particularly with some classes of drugs such as sodium-glucose cotransporter 2 (SGLT2) inhibitors and glucagon-like peptide-1 (GLP-1) receptor agonists, which act through specific endocrine or renal pathways [8, 10].

More recently, it has been suggested that upstream stress-response and metabolic signaling pathways may be considered potential therapeutic targets for T2DM because they are positioned closer to the molecular origins of the disease, including endoplasmic reticulum (ER) stress, inositol requiring enzyme 1-alpha/x-box binding protein-1 (IRE1α/XBP1) signaling, c-Jun N-terminal kinase (JNK)-mediated insulin receptor substrate (IRS) impairment, and reduced phosphoinositide 3-kinase/AKT (PI3K/AKT) activity [11–13]. Targeting such upstream pathways may make it possible to simultaneously modulate several disease-relevant mechanisms, including insulin resistance, β-cell stress, inflammatory signaling, altered lipid metabolism, and impaired adaptive stress responses, rather than merely lowering glucose after metabolic dysfunction is already established [14–17].

The IRE1α/XBP1 pathway is an evolutionarily conserved pathway, and a component of the unfolded protein response (UPR), which is activated in response to persistent ER stress. It links insulin signaling, ER stress, and metabolic regulation [18, 19]. Moreover, its involvement in impairment of β-cell function and eventual death, inflammation as well as insulin resistance, which are all key factors in T2DM pathophysiology, has made it an appealing therapeutic target for T2DM [20, 21]. Like the other branches of the UPR, IRE1α signaling is initially activated as an adaptive response aimed at restoring ER proteostasis and cellular homeostasis, principally through the splicing of XBP1 mRNA and subsequent induction of genes involved in protein folding, ER-associated degradation and secretory capacity. However, when ER stress is severe, persistent or unresolved, IRE1α signaling can shift towards maladaptive outputs, including regulated IRE1-dependent decay (RIDD) and pro-apoptotic signaling, thereby contributing to cellular dysfunction and cell death [22, 23]. Therefore, understanding the IRE1α arm of the UPR may provide an understanding of the means to either activate/enhance its adaptive response or inhibit the maladaptive outputs, with the hope of providing new therapeutic opportunities to prevent and manage T2DM.

The conserved nature of the IRE1α pathway also underlines its attractiveness as a therapeutic target, as preclinical studies on its therapeutic relevance can be more easily extrapolated to humans [24]. This is so because experimental animals often possess orthologous proteins that operate through comparable molecular mechanisms to humans. Consequently, modulating such a pathway in a preclinical model can reveal mechanistic relationships that are also operative in humans [25, 26]. This review highlights the implications of unresolved ER stress in the pathophysiology of T2DM, the evolutionarily conserved nature of the IRE1α/XBP1 signaling pathway and its crosstalks with other relevant pathways implicated in T2DM, which all makes it worth considering as a pharmacological target for T2DM.

## Endoplasmic reticulum stress/Unfolded protein response (ER stress/ UPR)

The UPR pathway is under the moderation of three major ER transmembrane stress sensors [27]. Under basal conditions, the ER chaperone glucose-regulated protein-78 (GRP78), also known as BiP, associates with the ER luminal domains of the three canonical ER stress sensors: inositol-requiring enzyme 1 alpha (IRE1α), protein kinase R-like ER kinase (PERK), and activating transcription factor 6 alpha (ATF6α). This association keeps the sensors inactive. During ER stress, GRP78 detaches from IRE1α and PERK as it binds unfolded proteins [28]. This leads to dimerization and trans-autophosphorylation of IRE1α and PERK, thereby activating their cytosolic enzymatic domains. It was also earlier suggested that unfolded proteins may directly activate IRE1α by binding to its luminal site [29]. ATF6α follows a different activation route upon dissociation from GRP78, as it moves from the ER to the Golgi body, where it is cleaved by site-1 and site-2 proteases to release an active N-terminal transcription factor [23, 30].

Together, the three ER membrane stress sensors and their downstream proteins attempt to re-establish proteostasis in the ER via enhanced folding capacity, reduced demand for protein folding, and enhanced degradation of terminally misfolded proteins, but persistent activation shifts the UPR from adaptation toward inflammation and apoptosis [30, 31]. Growing evidence also indicates that IRE1α signaling branch is a key regulator of metabolic homeostasis [32, 33]. The IRE1α signaling pathway has been recognized as an important regulator of metabolic homeostasis, connecting ER stress to glucose metabolism, lipid regulation, and pancreatic β-cell survival [34].

## IRE1α/XBP pathway

The IRE1α/XBP1 pathway serves as a signaling node that involves ER metabolic homeostasis, inflammatory and survival pathways [35]. Its activation and downstream signaling are key processes through which chronic ER stress is linked to pathophysiology of T2DM. IRE1α consists of a stress-sensing domain on the luminal side as well as kinase and endoribonuclease (RNase) domains on the cytoplasmic side [21]. Accumulation of unfolded proteins during ER stress leads to the detachment of GRP78 from IRE1α. This exposes the luminal domain and allows IRE1α to oligomerize and trans-autophosphorylate, thereby activating its RNase domain [21]. Once activated, IRE1α triggers downstream signaling events that contribute to both adaptive and maladaptive stages of the UPR [36].

One of the most well-characterized functions of activated IRE1α is the unconventional splicing of XBP1 mRNA [37]. IRE1α cleaves a 26-nucleotide intron from XBP1 mRNA in the cytoplasm, resulting in a translational frameshift that produces the spliced XBP1 (XBP1s) [37]. XBP1s is a potent basic leucine zipper (bZIP) transcription factor that moves to the nucleus where it regulates the expression of genes involved in ER-associated protein folding and quality control, ER biogenesis and expansion, lipid biosynthesis and membrane remodeling as well as secretory pathway enhancement [38]. Through these functions, XBP1s restores ER capacity and promotes cellular adaptation to stress. However, under chronic activation, sustained IRE1α signaling can extend beyond adaptive responses and contribute to maladaptive pathological signaling networks [21].

The conserved nature of the IRE1α/XBP1 signaling pathway [39, 40] has important consequences on biomedical research. Its involvement in diverse physiological processes including immunity [41], lipid homeostasis [42], cell survival, insulin resistance, β-cell death and protein secretion [43], , makes the pathway suitable for providing mechanistic explanation for how its dysregulation contributes to diseases. As the pathway constitutes a vital and universal component of cellular physiology, having similar functions in different species, findings from model organisms might be more easily extrapolated to human disease [16]. This makes the pathway highly relevant as a therapeutic target. It was posited that drugs which modulate the IRE1α/XBP1 signaling pathway could potentially influence critical metabolic and stress-response processes that contribute to the pathophysiology of T2DM [16].

## Relevance of IRE1α/XBP1 pathway in metabolically active tissues: adaptive responses and maladaptive outputs

Metabolically active tissues, including the liver, adipose tissue, pancreatic islets and skeletal muscle, continually adjust their metabolic and secretory activities in response to nutritional, biochemical and hormonal signals. The IRE1α/XBP1 pathway is important in these tissues because it links endoplasmic reticulum (ER) capacity with metabolic regulation [37, 44].

The liver best illustrates the relationship between proteostasis and nutrient metabolism. XBP1 contributes directly to hepatic lipid regulation, as liver-specific XBP1 deletion reduces *de novo* fatty-acid synthesis and concentrations of circulating triglyceride and cholesterol [45]. During prolonged fasting, hepatic IRE1α-XBP1 signaling also promotes fatty-acid oxidation and ketogenesis through activation of PPARα [46]. The pathway additionally regulates hepatic glucose metabolism via XBP1s-mediated suppression of gluconeogenesis by interacting with FOXO1 and promoting its proteasomal degradation, thereby improving glucose tolerance [18]. In contrast, fasting-associated glucagon signaling activates protein kinase A, which phosphorylates hepatic IRE1α at Ser724 and enhances gluconeogenic gene expression independently of XBP1s [47]. During refeeding, XBP1s supports reduced hepatic glucose output and increased glucose utilization partly through UDP-galactose-4-epimerase (GalE) [48]. Insulin-activated AKT further promotes XBP1 splicing during feeding, whereas fasting-associated increase in unspliced XBP1 (XBPu) enhances cAMP-responsive gluconeogenic activity [12].

**Fig. 1:**
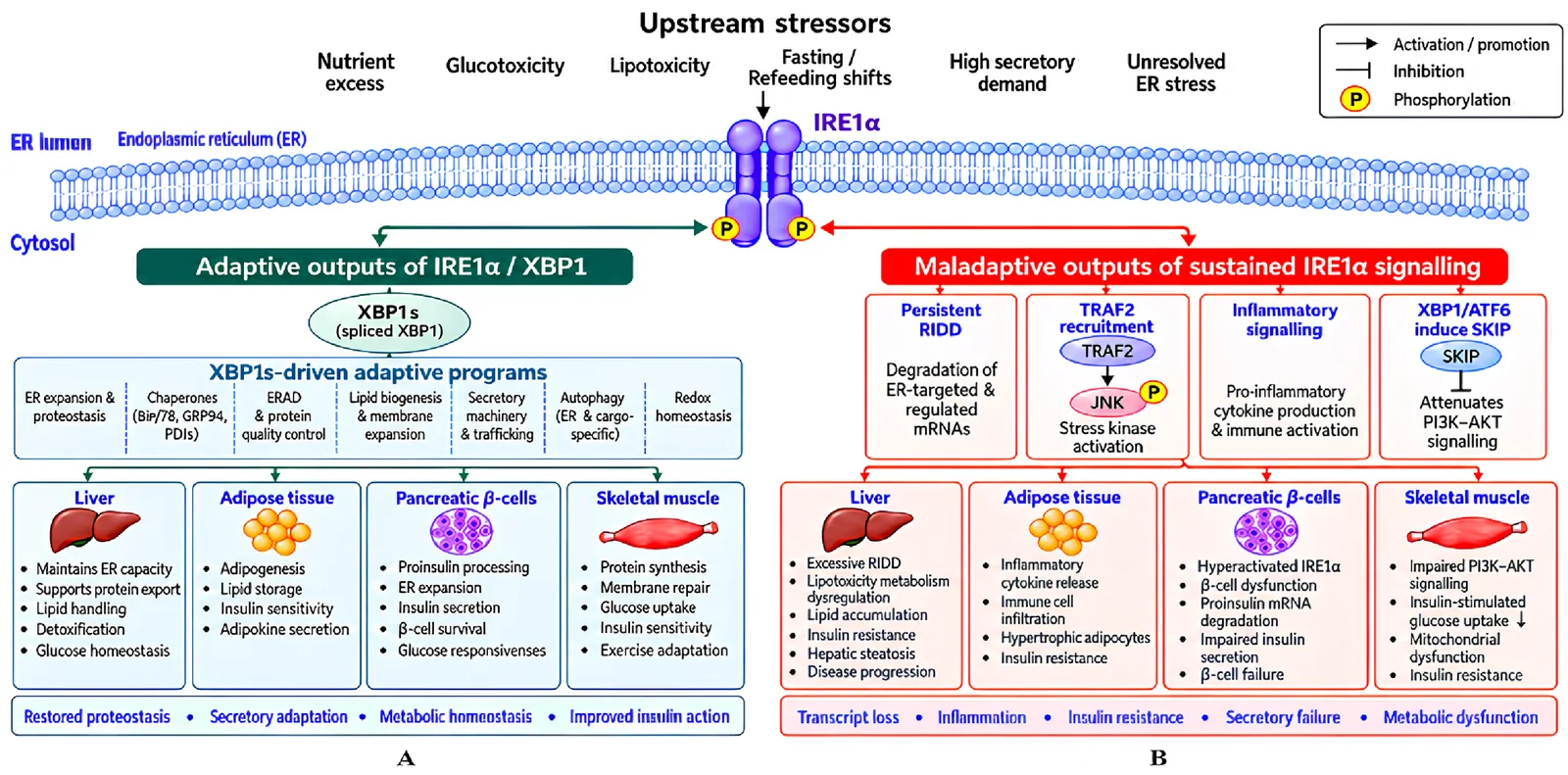
Adaptive responses and maladaptive outputs of the IRE1α-XBP1 pathway

In adipose tissue, IRE1α/XBP1 supports adipocyte differentiation and metabolic competence. CCAAT/enhancer-binding protein beta (C/EBPβ) induces XBP1s during adipogenesis, while XBP1s maintains adipocyte differentiation by promoting the expression of C/EBPα. Thus, disruption of either IRE1α or XBP1 impairs adipocyte differentiation [49]. The IRE1α/XBP1 pathway is also critical in β-cells, where glucose-induced IRE1α-dependent XBP1 splicing expands the cellular machinery required for proinsulin synthesis, processing and secretion, while limiting oxidative stress [19]. β-cell-specific XBP1 deficiency reduces insulin granules, disrupts proinsulin processing and weakens glucose-stimulated insulin secretion [50]. XBP1 also contributes to β-cell identity and compensatory function during obesity-related metabolic stress [51]. Although less extensively characterized in metabolic homeostasis, skeletal muscle also uses IRE1α/XBP1 signaling adaptively. The pathway is activated after muscle injury, and myofibre-specific deletion of IRE1α or XBP1 compromises muscle regenerative capacity and reduces abundance of satellite cells in a mice model of muscular dystrophy (Fig. 1A) [52].

The IRE1α/XBP1 pathway can become maladaptive when glucotoxicity, lipotoxicity or unresolved ER stress causes sustained or qualitatively altered IRE1α signaling (Fig. 1B). In this setting, the biological output may shift from predominantly XBP1s-mediated restoration of proteostasis to persistent regulated IRE1-dependent decay (RIDD), stress-kinase signaling and inflammatory responses. RIDD may initially be adaptive by reducing the burden of ER-targeted transcripts, but excessive RNase activity can deplete mRNAs required for specialized cellular functions. In the liver, for example, XBP1 deficiency causes compensatory IRE1α activation and increased RIDD-destruction of transcripts involved in lipogenesis and lipoprotein metabolism, profoundly altering systemic lipid homeostasis [22, 53, 54].

In β-cells, time-dependent activation of the IRE1α is especially relevant. Chronic hyperglycemia exposure hyperactivates IRE1α, suppresses insulin gene expression and promotes insulin mRNA degradation, converting an adaptive response into a secretory dysfunction [54]. Persistent ER stress also engages IRE1α-dependent recruitment of tumor necrosis factor receptor-associated factor 2 (TRAF2) and activation of JNK, creating a mechanistic link between ER stress, inflammatory signaling and impaired insulin action (Fig. 1B) [55]. This mechanism is relevant to obesity, in which ER stress is increased in metabolic tissues and JNK activation interferes with insulin receptor signaling, while reduced XBP1 function predisposes to systemic insulin resistance [56]. Maladaptive consequences are also evident in skeletal muscle. Under ER stress, XBP1, alongside ATF6, upregulates skeletal muscle kidney-enriched inositol polyphosphate phosphatase (SKIP), which attenuates PI3K-AKT signaling and insulin-stimulated glucose uptake, linking chronic UPR activation to muscle insulin resistance (Fig. 1B) [57].

Thus, the adaptive-to-maladaptive transition cannot be defined simply by activation of IRE1α. It depends on the magnitude and duration of activation, the relative generation and actions of XBP1s and unspliced XBP1 (XBP1u), the substrate selectivity of RIDD, and tissue-specific interactions with metabolic and inflammatory pathways. Inadequate IRE1α/XBP1 activity limits ER expansion, insulin secretion, lipid handling and tissue adaptation, whereas prolonged signaling can drive transcript loss, insulin resistance and secretory failure [58].

Glucotoxicity, lipotoxicity, fasting-refeeding cycle, high secretory demand, and unresolved ER stress converge on IRE1α. The adaptive XBP1s arm (A) targets proteostasis, ER expansion, chaperone and ERAD activity, secretory capacity, autophagy, and redox control across the liver, adipose tissue, pancreatic β-cells and skeletal muscle. This leads to adaptive outputs such as restoration of proteostasis, secretory adaptation, metabolic homeostasis and enhanced insulin action. Sustained IRE1α signaling (B) instead engages RIDD, TRAF2-JNK, inflammatory pathways, and impaired PI3K-AKT signaling, culminating in transcript loss, inflammation, insulin resistance, secretory failure, and metabolic dysfunction.

## IRE1α/XBP1 in the pathophysiology of type 2 diabetes mellitus

The IRE1α/XBP1 pathway is a critical molecular interface through which the diverse metabolic stressors associated with T2DM are translated into tissue-specific adaptive or pathological responses. In pancreatic β-cells, where exceptionally high demand for proinsulin synthesis imposes a substantial ER folding burden, glucotoxic and lipotoxic stress leads to β-cell dysfunction [54, 59]. Beyond the β-cells, obesity-associated ER stress in the liver and adipose tissue activates stress kinases, particularly JNK, which impair insulin receptor signaling and contribute to systemic insulin resistance, conversely, impaired XBP1 activity increases susceptibility to insulin resistance [56]. ER stress/UPR is thus implicated in the main underlying pathogenic mechanisms of T2DM, including insulin resistance and pancreatic β-cell dysfunction [43].

### IRE1α-XBP1 in insulin resistance

**Fig. 2:**
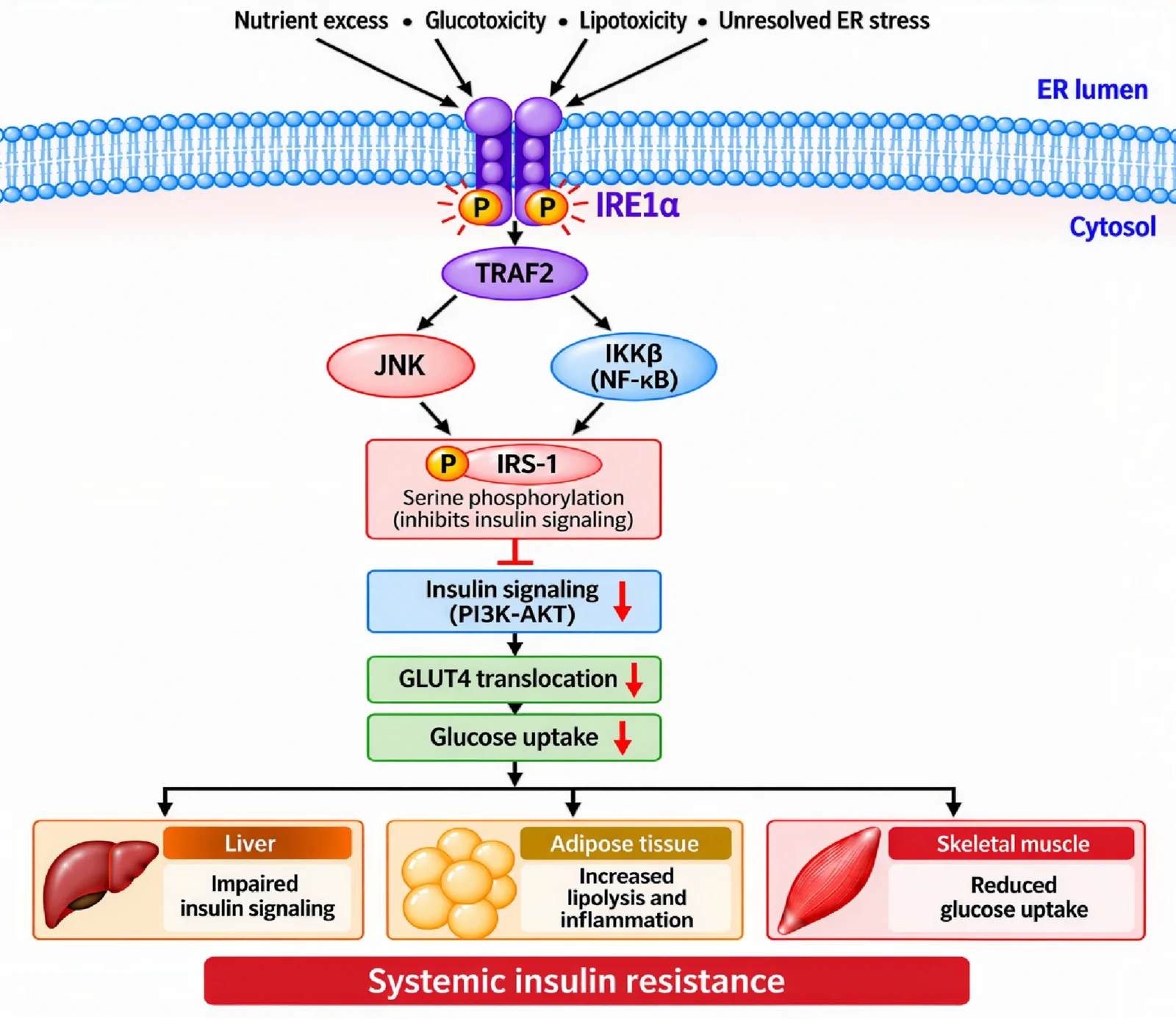
Cellular stressors and unresolved ER stress lead to dysregulated insulin signaling and systemic insulin resistance

Activation of the UPR signaling for an extended period is a major driver of insulin resistance. Under conditions of prolonged excess nutrient, UPR sensors trigger stress-related kinase, JNK [56]. Constant activation of the IRE1α pathway enhances JNK signaling, which leads to phosphorylation of insulin receptor substrate-1 (IRS-1) on serine residues rather than tyrosine residues, thereby inhibiting activation of IRS-1. This disrupts downstream insulin signaling including PI3K recruitment and activation of AKT [60], thereby decreasing insulin-stimulated glucose entry into insulin-sensitive tissues (Fig. 2). Earlier findings indicated that ER stress also impairs insulin sensitivity by delaying the maturation and delivery of newly synthesized insulin receptors from the endoplasmic reticulum to the cell surface [61].

ER stress also activates the nuclear factor-kB/inhibitor of nuclear factor kappa-B kinase (NF-kB/IKKβ) and other stress-sensitive inflammatory pathways. Cytokine and chemokines produced in liver and adipose tissue experiencing ER stress further stimulates serine phosphorylation of IRS, sustain macrophage recruitment and intensify low-grade metabolic inflammation [62, 63]. Defective insulin suppression of lipolysis in insulin-resistant adipose tissue elevates circulating free fatty acids. These lipids generate diacylglycerols, ceramides and oxidative stress, which reinforce ER stress and inhibit insulin receptor signaling in liver and muscle, further creating a feed-forward cycle between lipid overload and insulin resistance (Fig. 2) [64]. Prolonged activation of UPR ultimately disrupts insulin action and promotes systemic insulin resistance [65], thereby establishing ER stress as a critical molecular factor in impaired insulin signaling in T2DM.

Glucotoxicity, lipotoxicity, and unresolved ER stress activate IRE1α and recruit TRAF2. JNK and IKKβ-NF-κB signaling then converge on inhibitory serine phosphorylation of IRS-1, suppressing PI3K-AKT signaling. Reduced PI3K-AKT activity decreases GLUT4 translocation to the membrane and glucose uptake. At tissue level, the pathway is associated with impaired hepatic insulin signaling, increased adipose-tissue lipolysis and inflammation, and reduced skeletal-muscle glucose uptake. These coordinated defects culminate in systemic insulin resistance.

### IRE1α-XBP1 pathway and pancreatic β-cell dysfunction

Insulin biosynthesis in pancreatic β-cells begins with synthesis of preproinsulin on rough ER ribosomes. The nascent preproinsulin then moves via the N-terminal attached signal peptide towards the ER membrane where synthesis continues while the growing preproinsulin chain is simultaneously being translocated into the ER lumen, a process known as co-translational translocation. The signal peptide is eventually removed, thereby producing proinsulin which undergoes folding and formation of the three disulfide bonds: two interchain disulfide bonds linking the A and B chains and one intrachain disulfide bond within the A chain. Correctly folded proinsulin is exported via the Golgi to secretory granules where convertases and carboxypeptidase E process it into mature insulin [66, 67].

Pancreatic β-cells rely on efficient UPR signaling because of the significant demand in insulin synthesis and secretion [43]. Under early metabolic stress, insulin synthesis is supported by the UPR-related increase in protein-folding capacity. The IRE1α-XBP1 pathway is especially important in this regard as it promotes oxidative folding of proinsulin by inducing the protein disulfide isomerase family members. However, chronic activation of the pathway due to prolonged ER stress decreases levels of proinsulin and insulin, as well as insulin secretion [68].

Accumulation of unfolded and misfolded proinsulin impairs formation of disulfide bonds as well as the ER-Golgi trafficking and secretory granule formation. IRE1α hyperactivation can also degrade insulin mRNA via RIDD [54]. Chronic ER stress can additionally activate stress-kinase pathways, including apoptosis signal-regulating kinase-1 (ASK1) and p38 mitogen-activated protein kinase (p38 MAPK). Furthermore, chronic ER stress may also lead to accumulation of abnormal proinsulin species and ultimately reduces the efficiency of β-cell to match insulin secretion to glucose demand [69]. The PERK/eIF2α pathway, in addition to limiting insulin biosynthesis, also promotes ATF4-CHOP-dependent β-cell apoptosis via oxidative stress, mitochondrial dysfunction and perturbations in cellular calcium handling [70]. These mechanisms impair proinsulin processing, decrease insulin biosynthesis, and reduce the functional β-cell mass over time [14, 71].

## Crosstalk of IRE1α/XBP1 with pathways relevant to the pathophysiology of type 2 diabetes

In addition to its canonical contribution in restoring endoplasmic reticulum (ER) homeostasis, the IRE1α/XBP1 arm of the unfolded protein response serves as a key signaling node that crosstalks with several other cellular pathways [23]. During prolonged or severe ER stress, IRE1α signaling extends beyond adaptive protein-folding and interacts with several other physiologically relevant pathways [72]. This broad crosstalk facilitates cellular coordination of metabolic and stress responses [73]. However, during chronic activation, this network of interaction facilitates the interdependence of molecular perturbations of T2DM mellitus [74].

### Crosstalk with Inflammatory Signaling Pathways

**Fig. 3:**
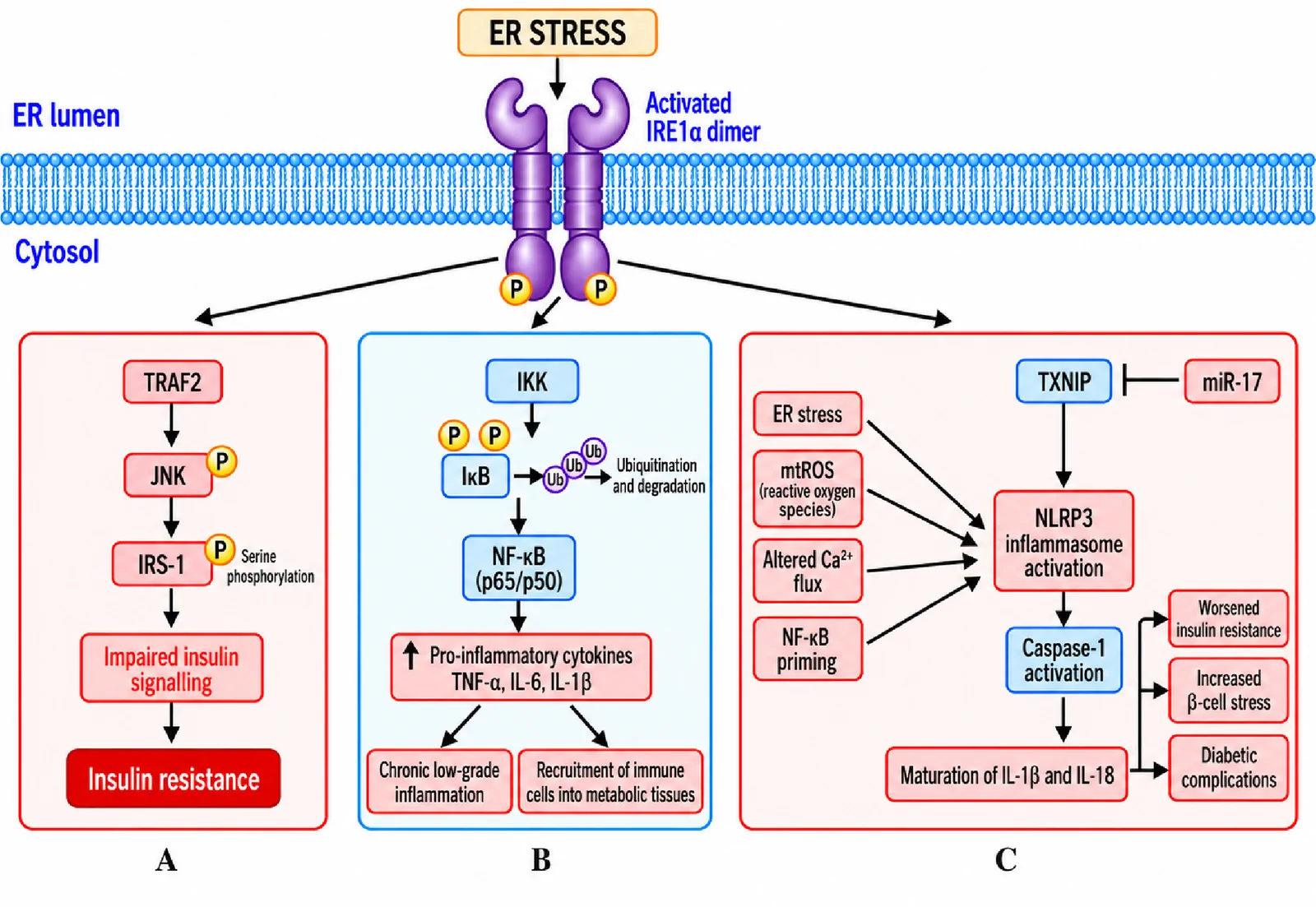
IRE1α crosstalk with inflammatory pathways in type 2 diabetes

IRE1α is a principal mediator that connects ER stress and inflammation [75]. Its cytosolic domain recruits tumor necrosis factor receptor-associated factor 2 (TRAF2) and activates JNK [55], while IRE1α/TRAF2 signaling can also activate nuclear factor kappa B/inhibitor of nuclear factor kappa B (NF-κB/IKK) signaling [62]. JNK activation promotes phosphorylation of IRS-1 on serine residues, thus disrupting insulin signaling (Fig. 3B) [60]. Moreover, NF-κB stimulation elevates the expression of pro-inflammatory cytokines, including tumour necrosis factor alpha (TNF-α), interleukin 6 (IL-6), and IL-1β which contributes to chronic low-grade inflammation in T2DM and recruitment of immune cells into metabolic tissues (Fig. 3B) [76]. Sustained ER stress also disrupts adipogenesis, adipokine production and lipid storage, with IRE1-XBP1, PERK and ATF6 branches all participating in adipose-tissue dysfunction [77].

Previous studies in mice and bone-marrow-derived macrophages have identified ER stress, mitochondrial reactive oxygen species (ROS), altered calcium flux and NF-κB priming as key inputs into activation of the NOD-like receptor family pyrin domain-containing 3 (NLRP3) inflammasome [78, 79]. Activation of the NLRP3 inflammasome promotes caspase-1-mediated processing of pro-IL-1β and pro-IL-18, thereby contributing to metabolic inflammation, insulin resistance, β-cell dysfunction and diabetic tissue injury [80]. IRE1α provides a direct mechanistic link between ER stress and NLRP3 activation through induction of thioredoxin-interacting protein (TXNIP). Under unresolved ER stress, hyperactivated IRE1α decreases the level of the TXNIP-repressive microRNA-17 (miR-17), stabilizing TXNIP mRNA and promoting TXNIP-dependent NLRP3 activation, caspase-1 cleavage and IL-1β secretion (Fig. 3C) [81]. IRE1α may additionally reinforce inflammasome signaling through IRE1α-TRAF2-dependent JNK and NF-κB pathways, mitochondrial stress, and excessive RNase/RIDD activity that amplifies terminal ER stress [55, 82]. The crosstalk between IRE1α and inflammatory pathways mediated by key proteins involved in inflammatory signaling and implicated in the pathophysiology of type 2 diabetes is illustrated in Fig. 3.

First, the IRE1α-TRAF2-JNK axis promotes IRS-1 serine phosphorylation, impaired insulin signaling, and insulin resistance (A). Second, IKK-dependent IκB degradation activates NF-κB and increases TNF-α, IL-6, and IL-1β, supporting chronic low-grade inflammation and immune-cell recruitment (B). Third, ER stress, mitochondrial ROS, altered Ca2 + flux, NF-κB priming, and TXNIP converge on NLRP3 inflammasome activation. Caspase-1 subsequently promotes maturation of IL-1β and IL-18, linking sustained ER stress to worsened insulin resistance, increased β-cell stress, and diabetic complications (C).

### Crosstalk of IRE1α with insulin signaling

**Fig. 4:**
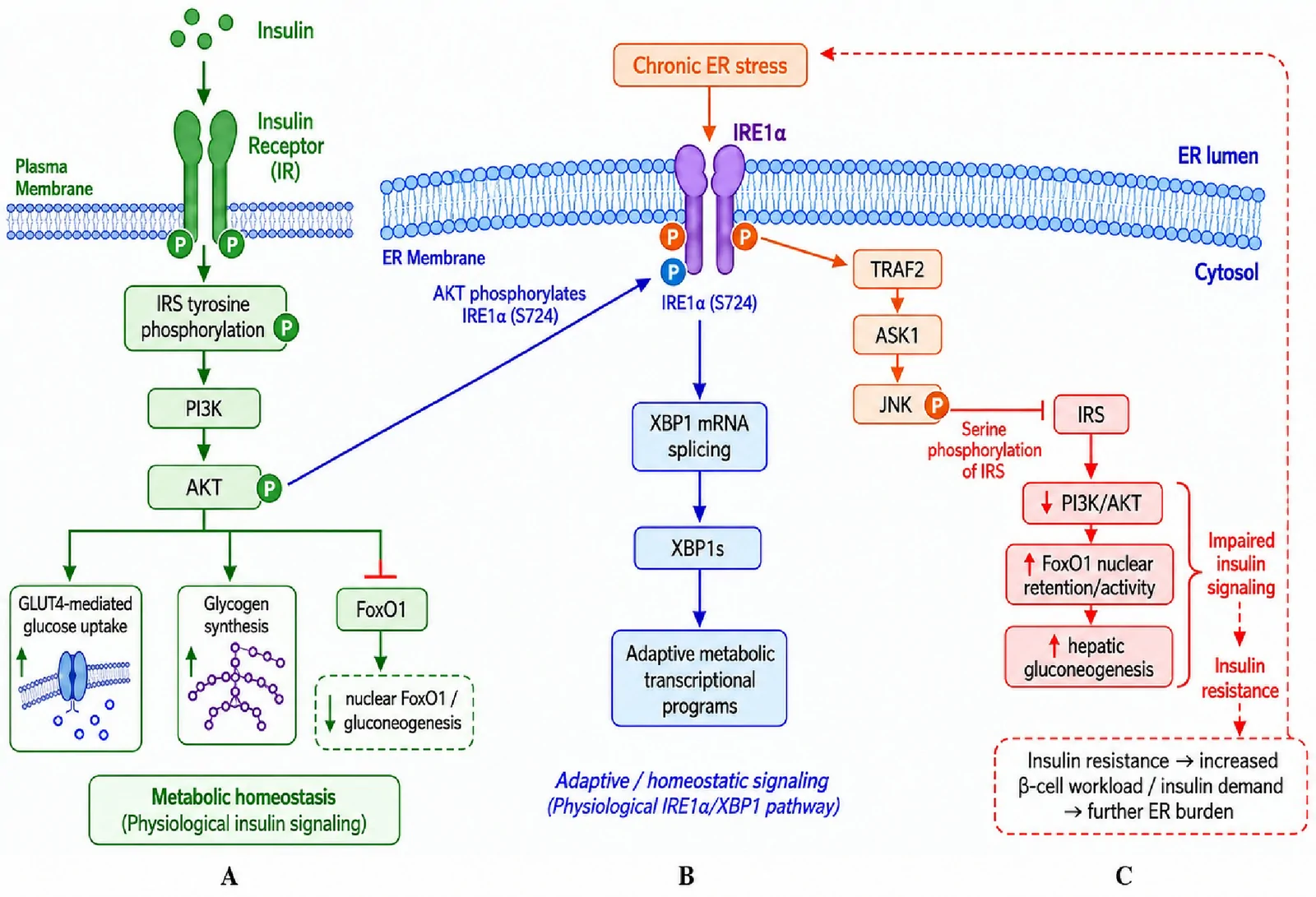
Crosstalk of IRE1α with physiological (tyrosine phosphorylation on IRS) and pathological (serine phosphorylation on IRS) insulin signaling

A major metabolic interface between IRE1α/XBP1 pathway and insulin signaling is the insulin receptor substrate (IRS)-PI3K-AKT-FoxO axis. In healthy insulin-responsive tissues, insulin receptor activation promotes IRS phosphorylation, PI3K recruitment, AKT activation, glucose uptake via GLUT4, glycogen synthesis and inhibition of hepatic gluconeogenesis (Fig. 4A). Dysregulation of this pathway is a defining molecular feature of obesity-associated insulin resistance and T2DM [11, 13]. IRE1α/XBP1 intersects this axis in two ways. First, physiological insulin signaling can activate the canonical IRE1-XBP1 pathway in the liver, allowing the postprandial insulin state to influence metabolism of glucose and lipid through transcriptional programmes that depend on XBP1 [12]. Also, AKT, activated by insulin directly phosphorylates IRE1 at S724 to effect the splicing of the XBP1 gene, thereby generating spliced XBP1s [12]. This observation indicates that the IRE1-XBP1 pathway is a nutrient-responsive pathway, and not simply a marker of cellular damage (Fig. 4B).

Chronic ER stress undermines insulin action through stress-kinase signaling. Mechanistically, activated IRE1α couples ER stress to JNK signaling through the IRE1α-TRAF2-ASK1 node, thereby linking unresolved ER stress to inflammatory and metabolic dysfunction [55, 83]. Activated JNK inhibits insulin signaling by promoting serine phosphorylation of IRS proteins, which weakens insulin receptor-IRS coupling and reduces downstream PI3K/AKT activity [60, 84]. In obesity-associated insulin resistance, ER stress suppresses insulin receptor signaling through JNK hyperactivation and IRS-1 serine phosphorylation, suggesting that ER stress is a molecular bridge linking excess nutrient with T2DM [56]. Reduced PI3K/AKT signaling also permits greater FoxO1 nuclear retention and activity, thereby sustaining hepatic gluconeogenic events during insulin resistance [85]. This generates a feed-forward loop in which excess nutrient activates ER stress, IRE1α-JNK signaling blunts insulin action, insulin resistance increases β-cell workload, and increased insulin biosynthetic demand further aggravates ER burden and β-cell failure [15] (Fig. 4C). The implication of IRE1α in both physiological and pathological insulin signaling is illustrated in Fig. 4.

Insulin receptor activation stimulates IRS tyrosine phosphorylation, PI3K-AKT signaling, GLUT4-mediated glucose uptake, glycogen synthesis, and suppression of FoxO1-dependent gluconeogenesis (A). AKT phosphorylation of IRE1α at Ser724 is linked to XBP1 splicing and adaptive metabolic transcriptional programmes (B). In chronic ER stress, IRE1α activates TRAF2-ASK1-JNK signaling, promotes inhibitory IRS serine phosphorylation, reduces PI3K-AKT activity, and increases FoxO1-dependent hepatic gluconeogenesis. The resulting insulin resistance increases β-cell workload and ER burden, reinforcing the stress cycle (C).

### Crosstalk of IRE1-XBP1 pathway with oxidative stress signaling

The crosstalk of IRE1-XBP1 pathway and oxidative stress signaling is especially consequential in pancreatic β-cells because insulin biosynthesis requires intensive oxidative folding of proinsulin in the ER. Experimental evidence shows that glucose physiologically activates IRE1α-mediated XBP1 splicing to expand the beta-cell secretory apparatus and limit oxidative injury [19]. It was previously reported that loss of β-cell IRE1α increases oxidative stress and inflammation, however, antioxidant treatment partly rescues glucose intolerance, indicating that redox injury is not merely secondary but contributes causally to β-cell dysfunction [19]. Another study using Ire1α conditional knockout (CKO) mice and established insulinoma cell lines further demonstrated that IRE1α/XBP1 induces protein disulfide isomerase family members required for efficient oxidative proinsulin folding; disruption of this process reduces proinsulin folding, insulin content and secretion [68].

Lipotoxicity provides another means by which IRE1α/XBP1 intersects T2DM signaling. Saturated fatty acids alter ER membrane composition and can activate UPR signaling through membrane lipid saturation [86]. β-cell lipotoxicity is associated with defective insulin secretion, oxidative stress, mitochondrial dysfunction, inflammation and apoptosis [87]. XBP1s-dependent ER expansion and lipid-handling programmes may initially compensate for the lipid overload, however, sustained exposure shifts the balance toward JNK, NF-κB and RIDD-mediated injury [77].

A distinct redox-sensing function of IRE1α has also been demonstrated outside the canonical XBP1 pathway. Hourihan et al. [88] demonstrated in HepG2 cells that acute oxidative stress induces IRE1α sulfenylation, activates p38 in an IRE1α-dependent manner and increases nuclear factor erythroid 2-related factor 2 (NRF2) signaling. Inhibition or knockdown of IRE1α, p38 or ROS generation attenuated this antioxidant response. The study established an IRE1α–p38–NRF2 axis that is mechanistically separate from conventional ER-stress-induced XBP1 splicing. This finding is highly relevant to the diabetic liver, where chronic excess nutrient, hyperglycemia, fatty-acid overload and mitochondrial dysfunction increase ROS production while ER stress simultaneously activates the unfolded protein response. Although this complete IRE1α–p38–NRF2 sequence has not yet been directly demonstrated in pancreatic β-cells in vivo, its relevance is biologically plausible because p38-dependent NRF2 activation can promote antioxidant protection in pancreatic islet cells [89], and β-cell-specific NRF2 activity is required for adaptive β-cell survival, proliferation and mass expansion during high-fat-diet stress [90].

Chronic metabolic stress can convert the same interface into a pathogenic event. It was reported that in experimental diabetic nephropathy, high-glucose-induced ER stress activated an XBP1-HRD1-NRF2 axis. XBP1 increased expression of HRD1, an ER-associated degradation E3 ligase, which interacted with and ubiquitinated NRF2, thereby reducing NRF2 antioxidant protection and increasing susceptibility to ferroptosis-associated epithelial-mesenchymal transition in renal tubular cells [91]. Thus, persistent IRE1α/XBP1 activity may suppress NRF2-mediated cytoprotection when ER stress is unresolved.

## IRE1α/XBP1 pathway as a therapeutic target for type 2 diabetes

Currently available antidiabetic drugs have greatly improved glycemic management and, in the case of newer agents such as GLP-1 receptor agonists and SGLT2 inhibitors, have added cardiovascular, renal and weight-related benefits [7]. However, the fact that the IRE1α/XBP1 pathway occupies a strategic position at the intersection of ER proteostasis, oxidative stress, metabolic inflammation, insulin resistance and β-cell secretory adaptation, makes it an attractive therapeutic target for T2DM [13, 68, 75]. Moreover, the functional divergence between adaptive IRE1α-mediated XBP1 splicing, which expands ER folding and secretory capacity, and chronic or excessive IRE1α signaling, which activates RIDD, JNK-linked inflammatory signaling, inflammasome activation and cell death [19, 23], provides a strong rationale for developing drugs that selectively modulate either response [92, 93].

Preclinical evidence suggests that targeting the IRE1α/XBP1 pathway may offer several potential advantages over and complement currently available antidiabetic therapies because it affords intervention upstream of pathogenesis of T2DM at the level of ER proteostasis and metabolic stress, rather than only correcting downstream hyperglycemia after β-cell stress and insulin resistance have already ensued [33].

The therapeutic relevance of the pathway arising from enhancement of adaptive IRE1α/XBP1s signaling has produced substantial metabolic benefits in experimental diabetes and obesity. The mechanistic advantage of targeting the adaptive IRE1α/XBP1 pathway for drug development is clear in the pancreatic β-cells where insulin is produced as proinsulin and folded in the ER before secretion. Experimental evidence shows that transient IRE1α activation supports insulin biosynthesis because IRE1α/XBP1 signaling induces protein disulfide isomerase family members needed for efficient oxidative proinsulin folding [19, 68, 94]. This differs from insulin secretagogues or exogenous insulin, which increase insulin availability but do not necessarily repair the folding environment that sustains endogenous β-cell function.

Modest hepatic XBP1s overexpression reportedly improved hyperglycemia and glucose tolerance in insulin-resistant mice partly through FoxO1 degradation, indicating that XBP1s can bypass defective insulin signaling as well as augment ER proteostasis [18]. Likewise, p38 MAPK-mediated phosphorylation increased XBP1s nuclear translocation and activity. Hepatic activation of this mechanism reduced ER stress and restored euglycemia in severely obese diabetic mice [95]. In adipose tissue, XBP1s overexpression promoted ER chaperone expression and high-molecular-weight adiponectin multimerization, improving glucose tolerance and insulin sensitivity in lean and ob/ob mice [96].

Targeting the IRE1/XBP1s pathway may also provide multi-tissue benefits. Selective activation of the adaptive response of the IRE1α/XBP1 pathway with IXA4 in diet-induced obese mice reportedly reprogrammed hepatic transcriptome, leading to reduced glucose production and steatosis, improved systemic glucose metabolism and liver insulin action, and enhanced pancreatic function. These protective effects of IXA4 were not accompanied by activation of RIDD as might be expected [16]. This approach of enhancing the adaptive response of the pathway could complement existing drugs because it addresses multiple pathogenic mechanisms of T2DM diabetes including hepatic insulin resistance, β-cell secretory stress, and ER adaptive failure, within one stress-response network [16]. A further study to improve the activity of IXA4 in other tissues apart from the liver led to the discovery of IXA62, which also activated the IRE1/XBP1s pathway in the kidney and lungs. The compound increased glucose-stimulated insulin release in both rat insulinoma cells and in liver of mice [97].

Conversely, the IRE1α/XBP1 has been utilized as a direct pharmacological target in studies using kinase-inhibiting RNase attenuators (KIRAs) or other IRE1α RNase inhibitors. These studies utilize inhibition of the maladaptive outputs of IRE1α (its RNase activity and subsequent deleterious effects) as a mechanistic pathway through which antidiabetic or β-cell-protective agents might exert their effects [98–100]. Recently, Blanc et al. [101] demonstrated in mice that loss of the endogenous negative regulator of IRE1α, Bax inhibitor-1 caused excessive IRE1α RNase activity, NLRP3 inflammasome activation, apoptosis/pyroptosis, β-cell loss and hyperglycemia. Pharmacological RNase inhibition with STF-083010 reversed β-cell failure and normalized the metabolic phenotype. In obese mice with established insulin resistance, STF-083010 similarly improved glucose tolerance and insulin sensitivity while suppressing metabolically activated adipose-tissue macrophages and inflammation [17].

More recently, Rustamov et al. [102] showed that by inhibiting phosphorylation of IRE1α in both C2C12 cells and high fat diet-fed mice, KIRA8 restored the activity of sphingosine-1-phosphate lyase, whose inhibition is implicated in insulin resistance. In addition, KIRA8 improved AKT phosphorylation and glucose uptake in muscle cells, and improved glucose metabolism in experimental animals. In β-cell lipotoxicity models, exendin-4 and GLP-1/cAMP signaling were also linked to IRE1α/XBP1 or XBP1-AKT survival pathways, although these studies are better interpreted as pathway-mediation evidence than as direct IRE1α/XBP1 drug-target studies [99, 103].

Other studies support the pathway as a therapeutic target in T2DM diabetes and insulin resistance. Tang et al. [104] reported that polyphenol extract of *Punica granatum* flower improved glucose and lipid parameters in high-fat diet/streptozotocin-induced T2DM diabetic rats, with the mechanism linked to AKT/GSK-3β activation and inhibition of the hepatic IRE1α-XBP1-CHOP stress axis. It was also demonstrated by Wu et al. [17] that pharmacological IRE1α RNase inhibition improved insulin sensitivity, glucose intolerance, thermogenesis, and adipose inflammatory remodeling in diet-induced obese mice.

**Table 1:** Classification of therapeutic strategies targeting IRE1/XBP1 signaling in type 2 diabetes

S/*N*	Therapeutic target / intervention	Experimental evidence and therapeutic effect	Reference
A	Enhancement of adaptive responses		
	Genetic/mechanistic proof of concept		
1.	Enhancement of hepatic XBP1s expression	Lowered serum glucose and improved glucose tolerance in insulin-resistant mouse models, partly by promoting FoxO1 degradation.	[18]
2.	p38 MAPK-mediated XBP1s phosphorylation and nuclear translocation	Enhanced hepatic XBP1s activity reduced ER stress and restored euglycaemia in severely obese diabetic mice.	[95]
3.	Adipocyte XBP1s overexpression	Increased ER chaperone expression and high-molecular-weight adiponectin multimerization; improved glucose tolerance and insulin sensitivity in ob/ob mice.	[96]
	Pharmacological modulation		
4.	Selective activation of IRE1/XBP1s by IXA4	Activated XBP1s without inducing RIDD or TRAF2/JNK; improved systemic glucose metabolism, hepatic insulin action, steatosis and pancreatic function in diet-induced obese mice.	[16]
5.	Selective activation of IRE1/XBP1s by IXA62	Activation increased glucose-stimulated insulin secretion in insulinoma cells and mice	[97]
B	Inhibition of maladaptive outputs		
6.	Inhibition of excess IRE1α RNase/RIDD–NLRP3 signaling by STF-083010	IRE1α RNase inhibition reversed β-cell failure, apoptosis/pyroptosis and metabolic dysfunction caused by excessive terminal UPR signaling in mice	[101]
7.	Inhibition of excess IRE1α RNase signaling in adipose tissue by STF-083010	Improved glucose tolerance and insulin sensitivity and reduced inflammatory/metabolically activated adipose macrophages in mice with established diet-induced obseity.	[17]
8.	IRE1α–S1PL dysregulation; KIRA8	Restored S1PL activity, Akt signaling and glucose uptake in muscle and improved glucose metabolism in high fructose diet-fed mice.	[102]

## Conclusion and future directions

Available evidence suggests that the IRE1α/XBP1 arm of the unfolded protein response is a biologically plausible and therapeutically attractive target in T2DM diabetes because it sits at the intersection of ER proteostasis, insulin signaling, inflammation, oxidative stress, lipid handling and pancreatic β-cell survival. Unlike many current antidiabetic drugs that primarily lower circulating glucose through downstream metabolic pathways, carefully regulated modulation of IRE1α/XBP1 could address earlier pathogenic events by improving adaptive ER capacity, supporting oxidative proinsulin folding, preserving glucose-stimulated insulin secretion, reducing maladaptive inflammatory and stress-kinase signaling, and limiting the transition from compensatory β-cell hyperactivity to β-cell exhaustion. The pathway is particularly promising because pre-clinical studies suggest that both activation and inhibition may be beneficial, depending on tissue, disease stage and degree of pathway activation (Table 1). These indicate that therapeutic success will require precision, rather than unregulated activation or inhibition. With continued investigation, the IRE1α/XBP1 axis holds significant promise as a next-generation therapeutic target for improving the treatment of T2DM.

Due to the intrinsic bidirectional nature of the IRE1α/XBP1 pathway and the consequent narrow therapeutic window, a major limitation in pharmacologically targeting the pathway for T2DM is attaining tissue-, dose-, duration-, and output-selective modulation of the pathway. For the IRE1α/XBP1 pathway to move from a molecular target to a clinically useful intervention for T2DM diabetes, studies are needed to elucidate the stage-specific and tissue-specific thresholds that differentiate adaptive IRE1α/XBP1 signaling from harmful hyperactivation. Comparison of selective XBP1s activation, IRE1α RNase attenuation and kinase-domain modulation in the same experimental models is also necessary. Moreover, determination of the ease of extrapolation of findings from animal and cell models to human T2DM diabetes is essential. It is also important to investigate the combination of IRE1α/XBP1 pathway modulators with established antidiabetic drugs, and the possibility that natural products or small molecules may modulate, rather than completely suppress IRE1α/XBP1 signaling.

## Data Availability

No datasets were generated or analysed during the current study.

## Abbreviations

Akt: Protein kinase B
AMPK: AMP-activated protein kinase
ASK1: Apoptosis signal-regulating kinase 1
ATF4: Activating transcription factor 4
ATF6: Activating transcription factor 6
BiP: Binding immunoglobulin protein
bZIP: Basic leucine zipper
C/EBP: CCAAT/enhancer-binding protein
CHOP: C/EBP homologous protein
eIF2α: Eukaryotic initiation factor 2 alpha
ERAD: Endoplasmic reticulum-associated degradation
FoxO: Forkhead box O1
GRP78: Glucose-regulated protein 78
HAC1: Homologous to ATF/CREB 1
HRD1: ER-associated degradation E3 ubiquitin ligase HRD1
IKKβ: inhibitor of nuclear factor kappa-B kinase beta
IRE1α: Inositol-requiring enzyme 1 alpha
IRS: insulin receptor substrate
IXA4: IRE1/XBP1s activator 4
JNK: c-Jun N-terminal kinase
KEAP1: Kelch-like ECH-associated protein 1
KIRA: Kinase-inhibiting RNase attenuator
MAPK: Mitogen-activated protein kinase
NF-κB: Nuclear factor kappa B
NLRP3: NOD-like receptor family pyrin domain-containing 3
NOD: Nucleotide-binding oligomerization domain
NRF2: Nuclear factor erythroid 2-related factor 2
p38 MAPK: p38 mitogen-activated protein kinase
PERK: Protein kinase RNA-like endoplasmic reticulum kinase
PI3K: Phosphoinositide 3-kinase
RIDD: Regulated IRE1-dependent decay
TRAF2: Tumor necrosis factor receptor-associated factor 2
TXNIP: Thioredoxin-interacting protein
UPR: Unfolded protein response
XBP: X-box binding protein
