# Correction: Targeting the IRE1α/XBP1 pathway in type 2 diabetes mellitus: from ER stress signaling to therapeutic opportunity

**DOI:** 10.1007/s11033-026-13041-w

**Published:** 2026-10-25

**Authors:** Olabisi Tajudeen Obafemi, Ademola Olabode Ayeleso, Bidemi Emmanuel Ekundayo, Blessing Ariyo Obafemi, Olusola Bolaji Adewale, Sogolo Lucky Lebelo, Monde Ntwasa

**Affiliations:** 1 Department of Life and Consumer Sciences, University of South Africa, Florida Campus, Johannesburg, 1709 South Africa; 2 Biochemistry Programme, College of Agriculture, Engineering and Sciences, Bowen University, Iwo, 232101 Nigeria; 3 Department of Biochemistry, Bamidele Olumilua University of Education Science and Technology, Ikere-Ekiti, Nigeria; 4 Department of Medical Biochemistry, Afe Babalola University, Km 8.5, Afe Babalola way, Ado-Ekiti, 360001 Ekiti State Nigeria; 5 Drosophila Research/Drug Metabolism and Toxicology Laboratories, Biochemistry Program, College of Sciences, Afe Babalola University, Km 8.5, Afe Babalola way, Ado-Ekiti, Ekiti, 360001 Nigeria

**Correction to: Molecular Biology Reports (2026) 53:1688**.

https://doi.org/10.1007/s11033-026-12876-7.

In this article the "Author contributions" statement was incorrectly given as 'OTO, AOA:Conceptualization, Writing – original draft. A.O.A, B.E.E: Conceptualization, Writing - original draft.B.A.O, O.B.A: Writing - original draft. S.L.L, M.N.: Writing – review and editing, Supervision' butshould have been 'OTO, AOA: Conceptualization, Writing – origi-nal draft. B.E.E: Conceptualization,Writing - original draft. B.A.O, O.B.A: Writing - original draft. A.O.A, S.L.L, M.N.: Writing – review andediting, Supervision'.

The original article has been corrected.

